# Optical Response of Silver Nanoneedles on a Mirror

**DOI:** 10.1007/s11468-015-9900-y

**Published:** 2015-02-25

**Authors:** Arjan Keeman, E. Stefan Kooij, Dick van Dam, Ruud E. I. Schropp, Marcel Di Vece

**Affiliations:** 1 Soft Condensed Matter, Debye Institute for Nanomaterials Science, Utrecht University, Princetonplein 5, 3584 CC Utrecht, The Netherlands; 2 Physics of Interfaces and Nanomaterials, MESA+ Institute for Nanotechnology, University of Twente, P.O. Box 217, 7500 AE Enschede, The Netherlands; 3 Nanophotonics—Physics of Devices, Debye Institute for Nanomaterials Science, Utrecht University, P.O. Box 80000, 3508 TA Utrecht, The Netherlands; 4 Solar Energy, Energy Research Center of the Netherlands (ECN), High Tech Campus Building 5, 5656 AE Eindhoven, The Netherlands; 5 Department of Applied Physics, Plasma & Materials Processing, Eindhoven University of Technology (TU/e), P.O. Box 513, 5600 MB Eindhoven, The Netherlands

**Keywords:** Silver, Nanoneedle, Ellipsometry, FDTD

## Abstract

Plasmonic properties of metal nanostructures are appealing due to their potential to enhance photovoltaics or sensing performance. Our aim was to identify the plasmonic characteristics of silver nanoneedles on a reflective layer in the polarized optical response. Experimental ellipsometry results are complemented by finite-difference time-domain (FDTD) calculations. Plasmon resonances on the nanoneedles can indeed be observed in the polarized optical response. This study reveals the details of the complex antenna-like behaviour of the nanoneedles which gives an agreement between experiment and FDTD simulation. The simulations show that the plasmon resonances lead to an effective negative refractive index, originating from the negative refractive index of the nanoneedles in combination with its supporting substrate, i.e. a mirror. This original study of a complex plasmonic system by ellipsometry and FDTD has great relevance for applications, making use of intricate light matter interaction.

## Introduction

Interest in “plasmonics”, the interaction of light with metal nanostructures, which merges optics with electronics [1, 2] at very small scales led to a wealth of studies in the past decade. A plasmon is the collective oscillation of an electron gas, which can be set in motion by an external electromagnetic field [3, 4]. The plasmonic response to electromagnetic fields opens a wide range of possible studies and applications, not in the least due to the much smaller size of plasmons as compared to the wavelength of light which limits traditional optical components (Rayleigh criterion) [5]. The interaction of an optical emitter with an electromagnetic field depends on its environment which can also have plasmonic structures [6–8]. For example, the luminescence intensity of an optical emitter can be enhanced or quenched by several orders of magnitude with concomitant changes in excited lifetime [9–11]. Early work in conjunction with surface-enhanced Raman spectroscopy (SERS) showed such photoluminescence enhancements with rough metal surfaces [12, 13]. The dimensions of metallic nanostructures as well as the dielectric environment determine the plasmon resonance frequency. At this frequency, the optical response is strongest which leads to the observation of absorption, local field enhancement, far-field scattering and plasmon guiding and coupling to optical modes [14]. The energy distribution between these effects depends strongly on the geometry, shape, size and distance of the nanostructures [15]. In photovoltaics, the implementation of such plasmonic nanostructures is currently explored with aiming at wave guiding, local field enhancement and increased scattering [16–20]. Scattering by the metal nanostructures [21] increases the light path length in a solar cell and is therefore an important mechanism to increase efficiency [22–24].

A free-standing metal nanowire within the solar cell is ideal as it combines the optimal light harvesting by scattering and efficient charge carrier extraction due to short electrical path length. Recent work on such structures in solar cells indeed provided increased efficiencies [25, 26].

A single resonance material with an anisotropy or chirality often has a negative refractive index [27–30]. Since the nanoneedle can be made with a changing diameter along its axis according to a cone, a large range of plasmon resonances is expected. Since anisotropic metal insulator structures have a negative refractive index associated with plasmon resonances, this makes the occurrence of a negative refractive index in a nanoneedle array plausible [30]. A negative value of the dielectric constant is obtained in the simple Drude model for frequencies below the plasmon resonance frequency *ω*_p_. Above *ω*_p_, the medium behaves as an ordinary medium with positive dielectric [31]. A negative magnetic permeability is obtained by the presence of magnetic moments induced by electrical currents in the metal nanostructure. This can be obtained by a nanowire array, where the nanowires are electromagnetically coupled to neighbouring ones.

Ellipsometry provides an experimental method to investigate the optical response of such structures. Although generally only specular information is obtained, plasmon resonances affect the differently polarized amplitudes and their relative phase, which makes it possible to detect the signature of a negative refractive index. Here we perform an ellipsometry experiment on silver nanoneedles on a flat bulk silver layer, i.e. a silver mirror. This structure is very complicated to model with traditional ellipsometry analysis schemes. Therefore we adopt an unconventional approach for ellipsometry, using finite-difference time-domain (FDTD) simulations to understand and interpret the results.

## Experimental

**Fig. 1:**
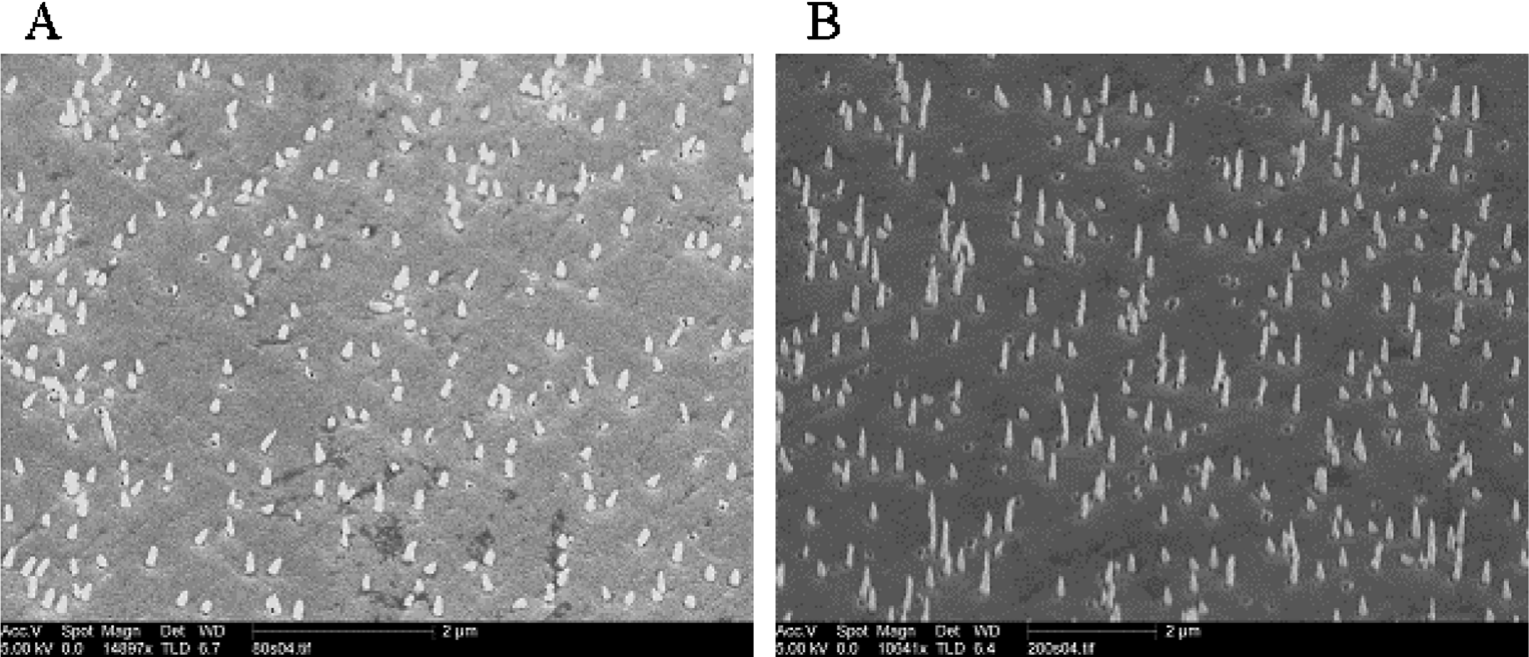
Scanning electron microscopy of silver nanoneedles on a silver layer. The needles are fabricated with a polycarbonate nanoporous membrane of **a** 80-nm- and **b** 200-nm-diameter holes

Silver nanoneedles are formed by thermal evaporation on polycarbonate nucleopore track-etch membranes (Whatman) with a pore size of either 80 or 200 nm. During the silver deposition, the pores are partially filled, giving rise to formation of silver nanoneedles within the pores, and a continuous film is formed on top of the membrane. Subsequently, the polycarbonate membrane was dissolved in chloroform after which the nanostructured silver film was transferred onto a glass substrate. The resulting nanoneedle structure is shown in Fig. 1. For the two different membranes used (with 80- and 200-nm pore diameters), we obtained nanoneedles with an average height of 270 ± 70 and 450 ± 150 nm and a base width of 100 ± 30 and 150 ± 30 nm, respectively. The nominal 80-nm pores are likely somewhat larger at the surface, resulting in the 100-nm base width. The fabrication method is relatively simple and as such enables a high reproducibility in production of the nanoneedle arrays.

The optical experiments were performed using a Woollam variable angle spectroscopic ellipsometer (VASE) system. Measurements were carried out as a function of photon energy in the range 0.75–4.5 eV with an energy step size of 0.1 eV; this corresponds to a wavelength range of 275–1650 nm. Spectra were obtained at incident angles in the range 45–75° with respect to the surface normal. In reflection ellipsometry, the change in the polarization state of light with a well-defined polarization upon reflection at an interface is measured. The complex reflection coefficient *ρ* is defined as

1 $$ \rho =\frac{r_{\mathrm{p}}}{r_{\mathrm{s}}}= \tan \varPsi \exp \left(\mathrm{i}\varDelta \right) $$

where *r*_p_ and *r*_s_ are the complex reflection coefficients for the parallel and perpendicular polarizations, respectively [32]. The amplitude ratio is expressed by tan *Ψ*, while *Δ* represents the phase difference.

FDTD calculations were performed with commercial software (Lumerical Solutions, Inc) on a cluster supercomputer consisting of several hundreds of multicore nodes of which ten were used for this study. 3D FDTD simulations were performed on a flat silver reference and two different nanoneedles with a height and width according to the average measured dimensions. The calculations were performed for s- and p-polarized light at angles of 45, 60 and 75°. The energy range between 0.8 and 4.5 eV included 15 data points chosen to optimize the comparison with the experiment. To simulate a fully periodic and infinitely uniform distribution of nanoneedles, we used Bloch boundary conditions in the direction of incident light, while in the perpendicular direction periodic (anti)-symmetric boundaries were used to reduce calculation time. The periodicity makes interaction between the nanoneedles possible. The simulation area had a length and width of 513 and 560 nm for the small and larger nanoneedles, respectively. These dimensions were in agreement with the nanoneedle density of the samples. The height varied between 473 and 2835 nm depending on wavelength. For small wavelengths, a smaller box was used, which reduced computation time. For the larger wavelengths to fit properly, a larger box size was required at the expense of computation time. Below the silver mirror and above the box, perfect matching layers (PML) were used to absorb the light. Advanced power absorption monitors were placed inside the silver structures [33]. Since the experimental sample was randomly covered by nanoneedles, the far-field projection was calculated after the simulation involving the nanoneedle array, from a single nanoneedle and its surrounding mirror. Considering the distances between neighbouring nanoneedles, the electromagnetic interaction between nanoneedles is expected to be negligible. As such, the present results provide information about the response of single/individual nanoneedles. This was achieved by a frequency domain power monitor above the plane wave source. Taking an array of nanoneedles would have increased the signal, but also would have created a phased array antenna which strongly enhances the signal in one direction, which does not represent the random distribution of the experimental sample. The pulse length of the incident plane wave was 50 fs with a band width of 8.825 THz. A convergency test was performed which confirmed sufficient accuracy with a smallest mesh of 2 nm^3^ on the nanoneedle.

## Results and Discussion

**Fig. 2:**
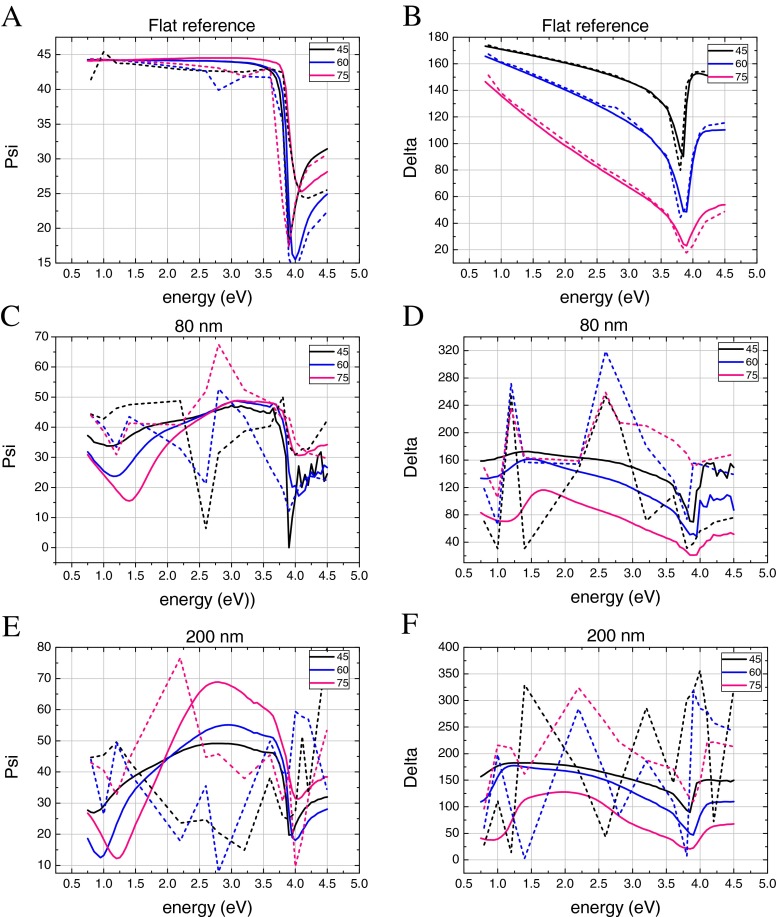
*Ψ* and delta values for different incident angles as a function of photon energy for **a**, **b** flat reference and **c**, **d** 80-nm and **e**, **f** 200-nm template holes. The *continuous lines* are experimental and the *dashed lines* are FDTD simulation values

Ellipsometry was performed on a flat silver reference and on the two nanoneedle samples as shown in Fig. 2. The *Ψ* and *Δ* spectra of the flat silver reference sample agree well with calculated spectra using the tabulated dielectric function values for silver in the literature. The features in *Ψ* and *Δ*, i.e. the minima near 3.8 eV, correspond to the bulk plasma frequency. At energies well below 3.8 eV, *Ψ* remains constant near 45° and does not depend much on energy and incident angle. In this energy range, the reflection of p- and s-polarized components are almost the same, giving rise to an amplitude ratio tan*Ψ* = 1; in this energy range, *Δ* exhibits a pronounced decline in this regime.

In contrast, the *Ψ* value as a function of energy of the spectra measured on nanoneedle samples has a similar form as the reference flat sample. At energies below approximately 3 eV the *Ψ* spectra exhibit minima at 1.4 and 1.25 eV for the short and long nanoneedles, respectively. As is evident from Eq. 1, a minimum in *Ψ* corresponds to a relatively small p-polarized component in the reflected spectra, as compared to the s-component. The s-polarized component is parallel to the substrate interface, while the p-polarized light has a component in the direction perpendicular to the substrate, and as such is sensitive to any absorption along the nanoneedle axis. Moreover, the magnitude of p-component of the polarized light increases for larger incident angles, in agreement with the deeper minima in *Ψ* in Fig. 2c, d. As indicated above, the energy minima in the infrared appear to correspond with the surface plasma frequency in the axial direction. The plasmon resonance frequencies of the nanoneedles depend on the nanoneedle length and aspect ratio. For example, Encina et al. [34] calculated for a silver nanowire of 480- or 320-nm long a resonance wavelength of 1805 nm (0.7 eV) or 1291 nm (1.0 eV). These dimensions are close to the ones used in this study. The *Ψ* spectra for the nanoneedles reveal values above 45° between the minimum for *Ψ* at low energies and the bulk plasmon resonance at high energies. This is most pronounced for the larger nanoneedles and corresponds to a stronger absorption of the s-polarized component, i.e. a plasmon resonance in the axial direction. The experimental *Δ* values also have a strong deviation from the flat reference at low energies. However, for the small nanoneedle, the minimum of the valley in *Δ* is 0.3 eV red shifted with respect to the valley in *Ψ*. A slight energy difference between the minima of *Ψ* and *Δ* also occurs in the bulk plasmon energy for the flat reference. The presence of peaks and valleys in the ellipsometry results reflects the fact that the nanoneedle is able to capture most of the incident light when it is at resonance. The absorption cross section is therefore much larger as compared to its geometric cross section. The ellipsometric parameters have also been calculated from FDTD simulations. The spectra for the flat reference sample agree remarkably well with the experimental result, validating the potential to use FDTD in simulating ellipsometric spectra.

### Small Nanoneedle (80-nm Hole: Height of 270 nm and a Base Width of 100 nm)

At the high-energy end, both *Ψ* and *Δ* from FDTD agree well with experiment. Although valleys at low energy for the small nanoneedle are also obtained in the FDTD simulation for 45 and 60° incidence, a blue shift of about 1 eV is observed in *Ψ* (Fig. 2c). One of the possible reasons may be that a mismatch between the experimental sample, which has a relatively broad dispersion in size and angle with respect to the substrate (Fig. 1), and the single nanoneedle used for FDTD. Within the large size dispersion, a particular size may have a stronger response and therefore shifts the plasmon resonance strength. At an incidence angle of 75°, no valley is present in the simulated *Ψ*. Since the plasmon resonance conditions for the nanoneedle in FDTD are precise and the energy resolution (between energy points) of the simulation limited, it is likely that the valley for this angle of incidence falls outside the energy range considered in the simulations.

The *Δ* values obtained using FDTD for the smaller nanoneedle (Fig. 2d) exhibits the same trend as its *Ψ* counterpart. The valley at low energies has been blue shifted with respect to the measurement by 0.7 eV. The FDTD simulation provides strong peaks in the *Δ* at about 2.5 eV which likely became smeared out in the experiment. For example, the 75° incidence (smaller nanoneedle) has a *Δ* peak value which is three times larger than the experiment and flat reference. This suggests that the phase of the reflected light changes strongly after incidence on a nanoneedle.

### Large Nanoneedle (200 nm Hole: Height of 450 nm and a Base Width of 150 nm)

The *Ψ* values of the FDTD simulation are considerably different from the experiment for all incidence angles (Fig. 2e). For 45 and 60° incidence, the FDTD simulations exhibit a maximum where the experimental results show a minimum and vice versa. It is very likely that again due to a mismatch between experimental and FDTD nanoneedle dimensions, a shift in plasmon resonance energy is responsible. However, the presence of extreme minima and maxima are both visible in experiment and simulation. At 75° incidence, the FDTD simulation deviates strongly from the other incidence angles: the position of minima and maxima are shifted. According to Hoffman et al. [35], this is a signature of negative refractive index. A minimum in *Ψ* corresponds to the Brewster angle, which can shift, approaching to zero. A negative refractive index reduces the s-polarized reflection (ideally nil). The strong difference as compared to the flat reference shows that the large nanoneedle has strong and multiple plasmon resonances.

Although the experimental *Δ* value has only a significant minimum at the low energies as compared to the flat reference, the FDTD simulations yield fluctuating *Δ* values (Fig. 2f). A minimum at 3.8 eV for both experiment and FDTD is present. It is probable that in the experiment, the fluctuations as obtained by FDTD are smeared out and result in a smoother curve. The FDTD results at 75° incidence have fewer fluctuations and agree rather well with experiment. Again, the difference of *Δ* for the different angle of incidence suggests a negative refractive index at 75°.

**Fig. 3:**
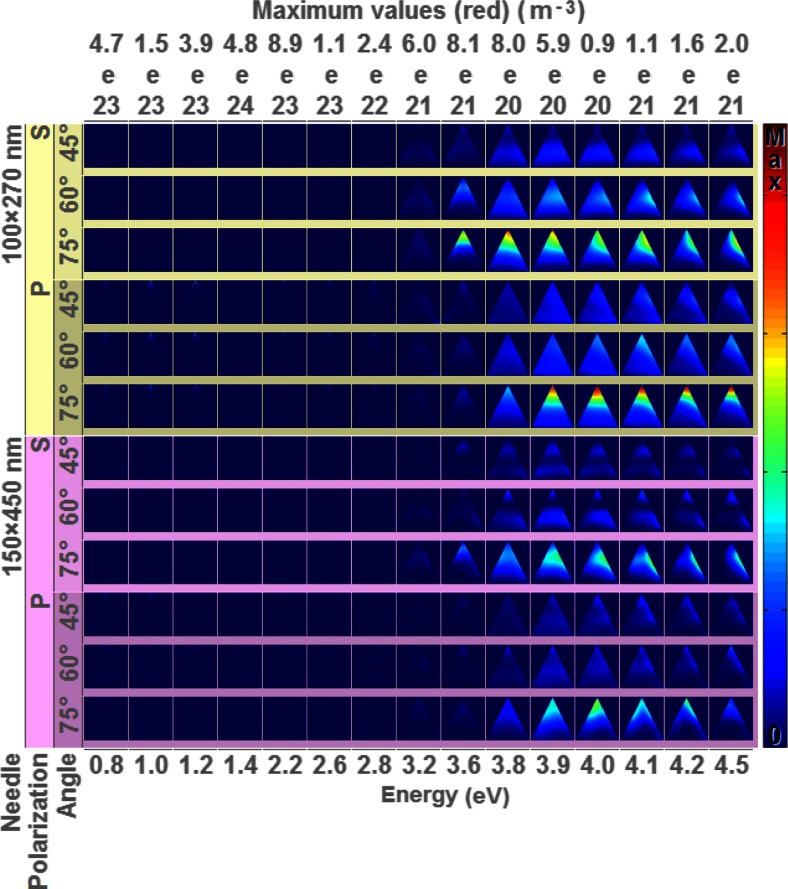
X-z cross-optical absorption cross section in the nanoneedle as a function of incident light energy (*x*-axis) for different polarizations and incident angle of the two different sizes (*y*-axis). The colour is proportional to the integrated optical absorption intensity in the silver nanoneedle. The height/width ratio of the nanoneedle is not to scale for clarity, and each column has equal scales, with the maximum scale value indicated for each column on the upper *x*-axis

The near-field response of the nanoneedles with incident light is shown in Fig. 3, which shows the optical absorption cross sections as a function of light energy for different polarizations. The colour, which is proportional to the integrated optical absorption intensity in the silver nanoneedle, varies considerably depending on the different conditions. The relatively blue colour of the nanoneedles at low energy is caused by the presence of a relatively high absorption at the tip of the nanoneedle due to strong local field enhancement. Since the colour scale has to include this high intensity, the remaining optical absorption in the nanoneedle becomes less pronounced. The clear border between high and low light absorption is positioned at 3.8 eV, which corresponds to the bulk plasmon resonance energy for silver. It is clear that at higher energies, light penetrates deep into the nanoneedle and therefore the optical absorption occurs throughout the volume of the nanoneedle. High absorption in the nanoneedle tip occurs at a high incident angle because the dimensions of the tip match the resonance conditions when aligned well with the light polarization. The exact position of the high absorption intensity spots depends on polarization and size of the nanoneedle, which set the plasmon resonance conditions.

At energies lower than the bulk plasmon resonance, light absorption is restricted to limited resonance conditions. These conditions occur mainly at the tip, since light at low energies cannot penetrate the nanoneedle main body. At these low energies, the strong, sometimes multiple, resonances of the near field at the tip affect the far-field behaviour as shown in the ellipsometry (Fig. 2) and far-field projections (Fig. 5). Plasmon resonances are particularly pronounced for p-polarized light as the electric field oscillates along the nanoneedle axis at various angles. Since the tip dimension of the nanoneedle is of the order of several tens of nanometre, much smaller than the distance between nanoneedles, electromagnetic interaction between nanoneedles is unlikely. This is confirmed by the optical absorption of single nanoneedles which can be explained by the incident light alone.

**Fig. 4:**
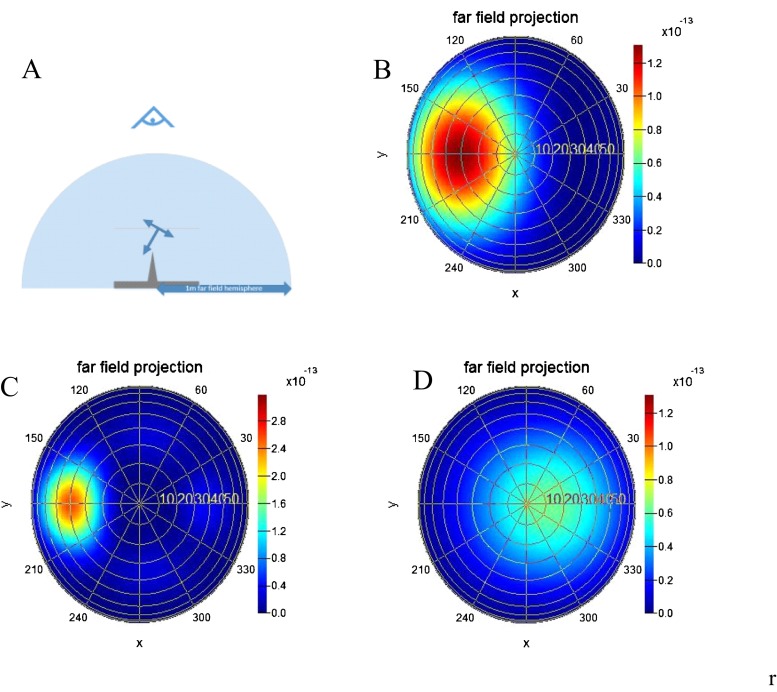
Schematic depiction of **a** geometry indicating that the nanoneedle orientation, incident light from right and far-field angular radiation pattern is measured. Far-field angular radiation patterns (V^2^/m^2^) for the large nanoneedle, 45° incidence, **b** s-polarized and 3.6 eV, **c** s-polarized 1.4 eV, and **d** p-polarized and 1.4 eV

The far-field angular radiation pattern is shown in Fig. 4 with the nanoneedle and far-field geometry (Fig. 4a). A typical response is shown in Fig. 4b where the returning light follows a specular direction. Due to the dependence of illumination angle spread on energy, the returning angle varies slightly. In Fig. 4c, a slight scattering profile can be distinguished by the somewhat higher intensity in the quadrants outside the specular reflection region. The symmetric lobe distribution over the four quadrants suggests a quadrupole mode, which is possible with s-polarized light on the nanoneedle at a diameter which results in retardation effects. A strong backward signal is observed for p-polarized light at 1.4 eV. The absence of light in the forward direction suggests strongly that light is experiencing a negative refractive index, resulting in this negative reflection. The complicated optical response of the nanoneedles is also responsible for the difference in specular reflection intensity between the flat reference and two nanoneedle sizes. With the nanoneedles present, a higher specular reflection intensity is obtained from the FDTD simulations. This can be explained by the phase relation after reflection on a nanoneedle which is more favourable for constructive interference than for the flat reference.

**Fig. 5:**
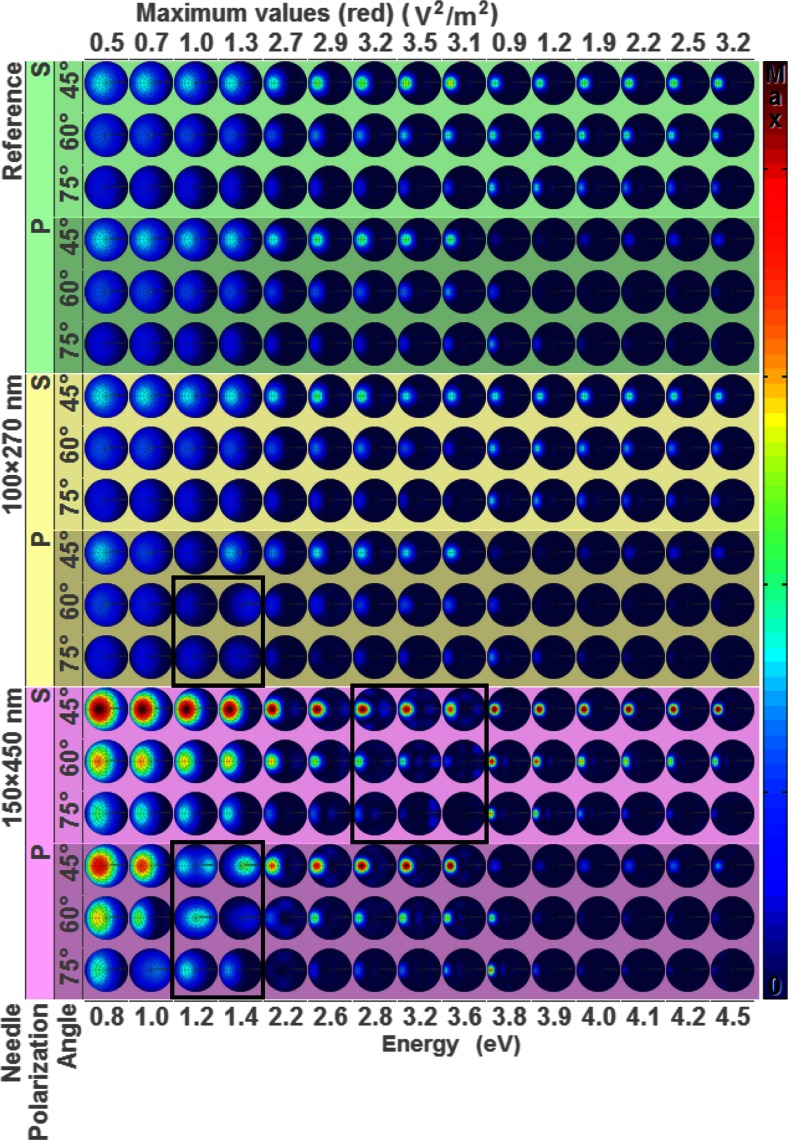
Angular radiation pattern as a function of photon energy for the flat reference and 80- and 200-nm hole template at different angles and polarization

In Fig. 5, the far-field radiation pattern is shown as a function of angle in each circle. The circles are plotted as a function of energy, incidence polarization and nanoneedle size. The bright spot in each circle corresponds to the returning light which has a larger size as compared to the experiment due to the Huygens principle, i.e. a wave front, on a very small sample. Since in the Huygens principle the amplitude scales inversely with the wavelength of light, the size of this intensity spot becomes smaller at higher energies. The reflected light intensity depends on energy, incidence angle and polarization. The angle of the reflected light increases upon lower energies. This is explained by considering the plate as a single slit source which generates a Fraunhofer pattern. The Fraunhofer pattern depends on the phase relation on the slit and therefore on the wavelength of the incident light. In most reflections, the returning light is in the specular orientation. However, at a few specific conditions, light is returned in another direction including its point of origin (indicated by black lined boxes). This effect is particularly strong for the larger nanoneedle and for p-polarized light around 1.1 eV. When light is reflected back to its point of origin, this indicates a negative reflection which can be explained by the occurrence of a negative refractive index in combination with a reflector. The energy at which this occurs is about 1.2 eV, which according to the ellipsometry as obtained by FDTD simulations, is lower than the plasmon resonance energy. Calculations show that this is a prerequisite for the occurrence of a negative refractive index with metallic nanostructures [30]. Negative refractive index materials are typically composed of a transparent medium with two interfaces [36]. Here we have a negative refractive index material composed of the nanoneedle layer with directly below it a reflective layer, the mirror. To understand how this combination produces a negative reflection needs a further detailed study.

## Conclusions

The ellipsometry experiment on silver nanoneedles on a mirror is explained by FDTD simulations. Plasmon resonances which are responsible for increased optical activity have been identified. The far-field response from simulations demonstrated reflections other than specular. At certain conditions with non-normal incidence, the reflection is directed towards the point of origin. This may point to the occurrence of a negative refractive index. The negative reflection as observed here by FDTD calculations needs further experimental confirmation.
